# Rapidly Progressive Cytophagic Histiocytic Panniculitis in a Child Triggered by Trauma and Scrub Typhus: A Dramatic Response to Cyclosporine

**DOI:** 10.7759/cureus.102680

**Published:** 2026-01-31

**Authors:** Pooja Unnikrishnan, Gopikrishna Mathurthi, Jami Vijayashree, Dilip Chandra

**Affiliations:** 1 Dermatology, Venereology, and Leprosy, Great Eastern Medical School and Hospital, Srikakulam, IND

**Keywords:** cyclosporine therapy, cytophagic histiocytic panniculitis, cytophagocytosis, pediatric panniculitis, scrub typhus–associated panniculitis

## Abstract

Cytophagic histiocytic panniculitis (CHP) is a rare and potentially life-threatening inflammatory panniculitis characterized by lobular lymphohistiocytic infiltration and cytophagic “bean-bag” histiocytes. Because its early features overlap with infectious panniculitis and hemophagocytic lymphohistiocytosis (HLH), diagnosis is often delayed, contributing to significant morbidity. We describe a rapidly progressive pediatric case of CHP triggered by antecedent trauma and scrub typhus infection.

A 10-year-old girl presented with seven days of high-grade fever and painful erythematous nodules over the extremities and trunk. Ten days prior, she had sustained a rope-induced traumatic ulcer on her leg, followed by persistent fever and progressive nodular lesions. Clinical examination revealed pallor, tachycardia, hepatosplenomegaly, and multiple tender subcutaneous nodules. Laboratory evaluation demonstrated severe anemia, thrombocytopenia, leukopenia, elevated liver enzymes, markedly raised inflammatory markers, and hyperferritinemia, with positive scrub typhus immunoglobulin M (IgM) serology and otherwise negative infectious and autoimmune workup.

Despite treatment with doxycycline, broad-spectrum antibiotics, and supportive care, the patient’s condition worsened. Skin biopsy revealed dense lobular panniculitis with numerous cytophagic histiocytes containing erythrocytes and nuclear debris, confirming the diagnosis of CHP. High-dose intravenous methylprednisolone was initiated, resulting in only partial clinical improvement. The subsequent addition of cyclosporine at a dose of 5 mg/kg/day led to rapid defervescence within 48 hours, the regression of nodules, and the normalization of hematological parameters.

This dramatic response highlights the therapeutic value of cyclosporine in severe or steroid-refractory CHP. This case underscores the importance of considering CHP in children presenting with febrile panniculitic nodules unresponsive to antimicrobial therapy, particularly when associated with cytopenias and systemic inflammation. Early dermatologic evaluation, timely biopsy confirmation, and the prompt initiation of immunosuppressive therapy are essential to prevent progression to HLH and ensure favorable outcomes. Our report adds to the limited pediatric literature on CHP and emphasizes the need for heightened clinical awareness and cyclosporine-based immunomodulation in rapidly progressive disease.

## Introduction

Cytophagic histiocytic panniculitis (CHP) is an exceptionally rare and potentially life-threatening inflammatory disorder characterized by lobular panniculitis with the infiltration of benign-appearing T lymphocytes and activated histiocytes exhibiting hemophagocytosis, classically described as “bean-bag cells” [1-4]. CHP may occur as an isolated cutaneous entity or in association with infections, autoimmune diseases, trauma, or malignancies, particularly subcutaneous panniculitis-like T-cell lymphoma (SPTCL) [3-6]. Pathogenetically, CHP is driven by immune dysregulation characterized by the excessive activation of cytotoxic T lymphocytes and macrophages, resulting in uncontrolled cytokine release and hemophagocytosis, which underlies its potential progression to hemophagocytic lymphohistiocytosis.

Clinically, CHP presents with painful subcutaneous nodules, fever, hepatosplenomegaly, lymphadenopathy, and progressive cytopenias. Systemic involvement may evolve into hemophagocytic lymphohistiocytosis (HLH), a hyperinflammatory syndrome associated with high mortality if not promptly recognized and treated [2,4,7]. Pediatric cases are especially uncommon, with fewer than 150 cases reported worldwide to date [1,3].

Infectious triggers such as Epstein-Barr virus (EBV), cytomegalovirus (CMV), human immunodeficiency virus (HIV), and bacterial infections have been implicated in the pathogenesis of CHP through immune dysregulation and macrophage activation [3,8,9]. Scrub typhus is a rare but increasingly recognized infectious trigger in endemic regions [10]. Early histopathological diagnosis and the timely initiation of immunosuppressive therapy, particularly corticosteroids and cyclosporine, are crucial to arrest disease progression and prevent evolution into HLH [5,6,11].

We report a pediatric case of rapidly progressive CHP triggered by antecedent trauma and scrub typhus infection, demonstrating a dramatic clinical and hematological response to cyclosporine therapy.

## Case presentation

A 10-year-old female child presented with a seven-day history of high-grade intermittent fever associated with chills and rigors, followed by the development of multiple painful erythematous nodules over the trunk and extremities. Ten days prior to presentation, the child sustained trauma to the left lower leg from a rope tied to an animal, after which a fluid-filled lesion developed.

Baseline laboratory investigations revealed pancytopenia with markedly elevated serum ferritin and D-dimer levels, while inflammatory markers remained normal (Table 1). Notably, despite severe systemic illness and evolving cytopenias, inflammatory markers, including erythrocyte sedimentation rate and C-reactive protein, remained within normal limits, a finding that may contribute to diagnostic delay.

**Table 1:** Laboratory investigations at presentation.

Parameters	Patient value	Units	Reference range
Hemoglobin (Hb)	4.5	g/dL	11.5-15.5
Red blood cell (RBC) count	1.6	million/µL	4.0-5.2
Total leukocyte count (TLC)	800	cells/µL	4,000-11,000
Platelet count	4,000	cells/µL	150,000-450,000
Erythrocyte sedimentation rate (ESR)	Normal	mm/hour	0-20
C-reactive protein (CRP)	Normal	mg/L	<5
Serum ferritin	971	ng/mL	7-140
D-dimer	2,740	pg/mL	<500
Total bilirubin	Elevated	mg/dL	0.2-1.2
Serum albumin	Decreased	g/dL	3.5-5.5
Prothrombin time (PT)	25.3	seconds	11-15
Bleeding time (BT)	7	minutes	2-7
Clotting time (CT)	10	minutes	5-11
Lipid profile	Normal	-	-
Bone marrow examination	Normal cytology	-	-
Scrub typhus serology	Positive	-	Negative

**Figure 1:**
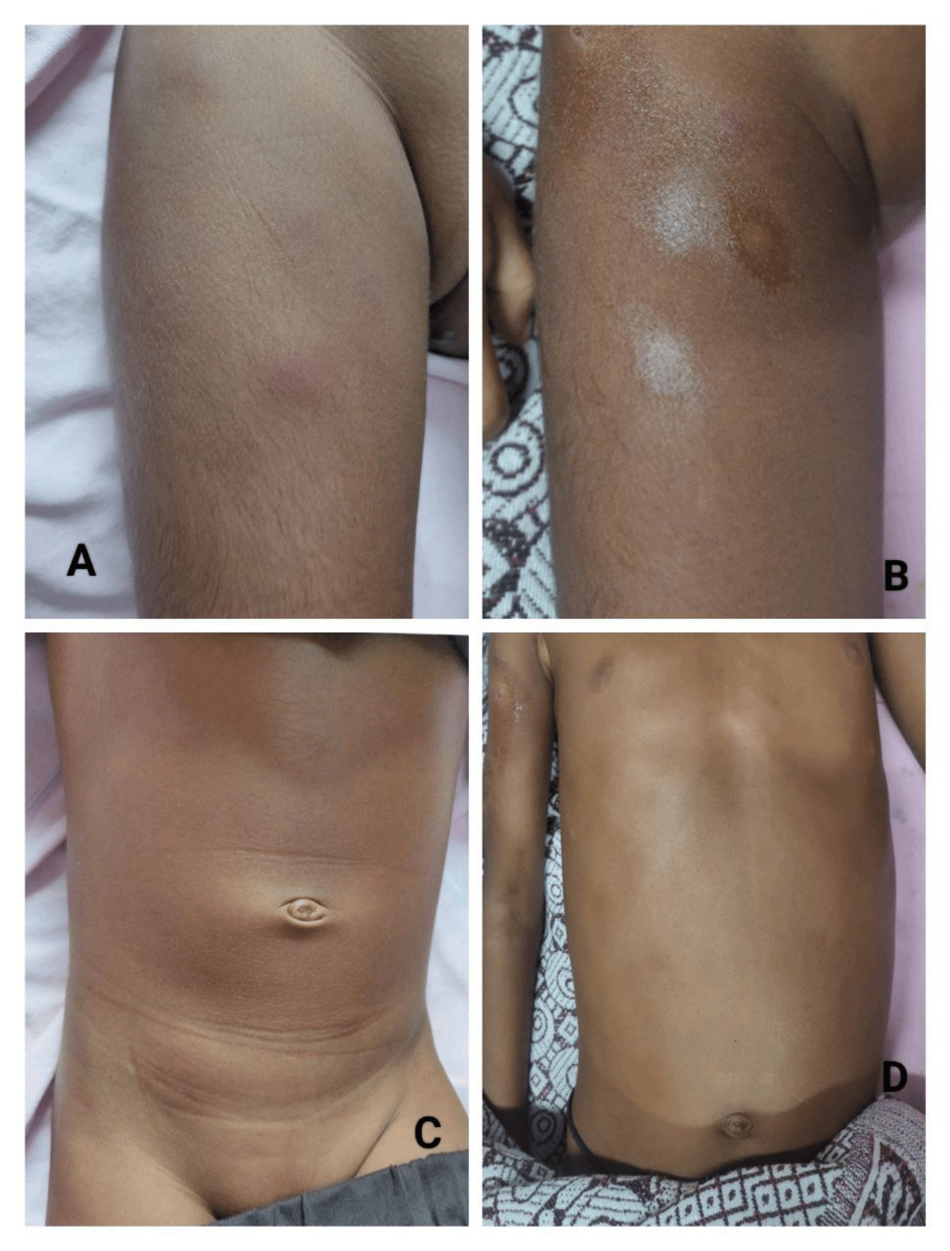
Clinical comparison of before to after treatment in cytophagic histiocytic panniculitis. (A) Tender erythematous subcutaneous nodule over the proximal lower limb at presentation. (B) Subcutaneous nodules over the lower limb began to regress after the initiation of immunosuppressive therapy. (C) Tender erythematous nodules over the trunk at presentation, representing active panniculitis. (D) Near-complete resolution of subcutaneous nodules and normalization of the skin following treatment with systemic corticosteroids and cyclosporine.

High-dose intravenous methylprednisolone (500 mg/day for three consecutive days) was initiated, followed by oral prednisolone and cyclosporine (50 mg/day) starting on day 5 of admission. Fever subsided within 48 hours of initiating immunosuppressive therapy, and the subcutaneous nodules began to regress. The progressive normalization of hematological parameters was observed. The complete healing of the ulcer and the near-total resolution of nodules were noted on follow-up (Figure 1A-1D).

**Figure 2:**
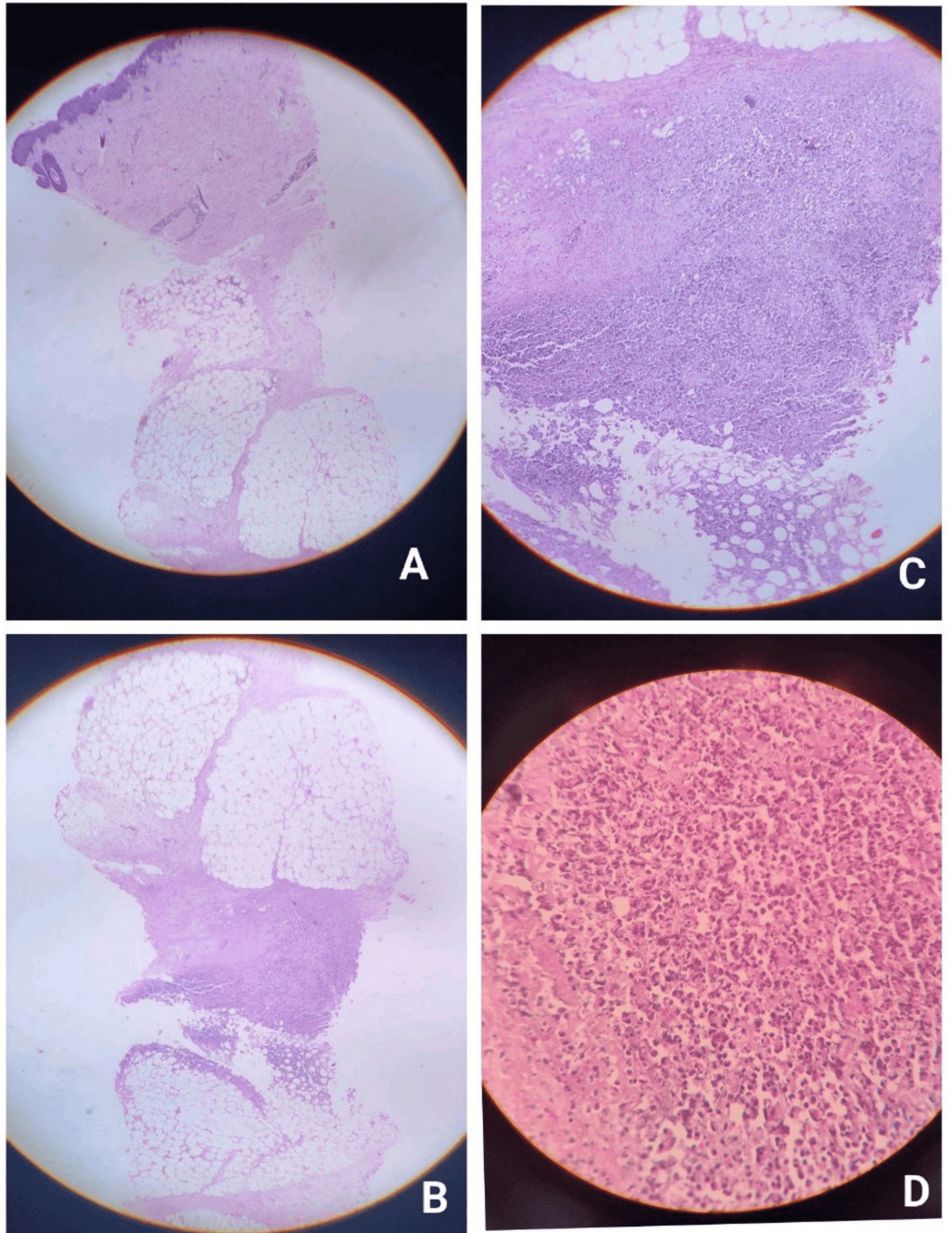
Histopathological features of cytophagic histiocytic panniculitis (hematoxylin and eosin stain). (A) Low-power view showing an intact epidermis with the involvement of the deeper dermis and subcutaneous tissue. (B) Low-power view demonstrating dense, patchy lobular lymphohistiocytic panniculitis involving the subcutaneous fat. (C) Intermediate-power view showing a moderately dense lymphohistiocytic infiltrate extending into the lower dermis with the prominent lobular involvement of the panniculus. (D) High-power view highlighting numerous large histiocytes with bilobed nuclei containing phagocytosed erythrocytes and nuclear debris (“bean-bag cells”), diagnostic of cytophagic activity.

The histopathological examination of a deep skin biopsy revealed an unaffected epidermis with dense lobular lymphohistiocytic panniculitis in the subcutaneous tissue. Numerous large histiocytes with bilobed nuclei containing phagocytosed erythrocytes and nuclear debris (“bean-bag cells”) were seen, along with the focal rimming of adipocytes by lymphocytes, confirming the diagnosis of cytophagic histiocytic panniculitis (Figure 2A-2D).

## Discussion

Cytophagic histiocytic panniculitis (CHP) is a rare autoinflammatory panniculitis with a variable clinical course, ranging from a self-limited cutaneous disorder to severe systemic disease with progression to hemophagocytic lymphohistiocytosis (HLH) [1-4]. Most reported cases, particularly in children and young adults, present with painful subcutaneous nodules, fever, hepatosplenomegaly, and cytopenias, closely resembling the clinical features observed in our patient [1-4]. Systemic manifestations and pancytopenia have been identified as predictors of severe disease and progression toward HLH, consistent with the early pancytopenia, hyperferritinemia, and coagulopathy noted in this case [1].

Unlike many previously reported cases, inflammatory markers such as erythrocyte sedimentation rate and C-reactive protein were normal despite significant systemic illness. This atypical finding has been described in isolated reports and may contribute to diagnostic delay, highlighting the need for clinical vigilance even in the absence of elevated inflammatory markers [3,5].

Immunopathogenetically, CHP is driven by the dysregulated activation of cytotoxic T lymphocytes and macrophages, resulting in excessive cytokine release and hemophagocytosis [2,7]. This mechanism explains the close biological relationship between CHP and HLH and supports the concept of a disease spectrum rather than distinct entities [2,7]. Although our patient exhibited features suggestive of evolving HLH, persistent fever, pancytopenia, hyperferritinemia, and coagulopathy, she did not fulfill the complete HLH-2004 diagnostic criteria. Bone marrow examination was normal, with no evidence of hemophagocytosis or neurological involvement, findings also reported in cases where hemophagocytic activity is initially confined to the skin [6,9].

Infectious triggers have been variably associated with CHP, with Epstein-Barr virus being the most commonly reported [2,4,8]. In this case, scrub typhus serology was positive, supporting its role as a potential immune trigger, particularly in endemic regions. Scrub typhus-associated macrophage activation and HLH have been previously documented, providing biological plausibility [10]. Other common infectious triggers, including Epstein-Barr virus and cytomegalovirus, were actively excluded. The presence of antecedent trauma further supports a multifactorial pathogenesis, as mechanical injury has been suggested as a local trigger for immune activation in CHP [3,6].

Histopathological examination remains pivotal for diagnosis. The characteristic findings of dense lobular lymphohistiocytic panniculitis with prominent hemophagocytosis (“bean-bag” cells) and the focal rimming of adipocytes in our patient are consistent with classical descriptions [3,4]. The absence of bone marrow involvement despite systemic manifestations emphasizes the importance of early deep skin biopsy in establishing the diagnosis and preventing delay [6,9].

Treatment outcomes in CHP depend on early and adequate immunosuppression. Delayed treatment or corticosteroid monotherapy has historically been associated with high mortality [2,4]. Increasing evidence supports the early use of cyclosporine, particularly in patients with systemic involvement. Studies by Hytiroglou et al. [5] and Crotty and Winkelmann [6] have demonstrated rapid remission and improved survival with cyclosporine, findings that parallel the dramatic response observed in our patient. Cyclosporine was administered at a dose of 5 mg/kg/day with close monitoring, and no adverse effects were observed.

Following the initiation of combined corticosteroid and cyclosporine therapy, the patient showed rapid defervescence, regression of panniculitic nodules, and progressive normalization of hematological parameters, including improvement in cytopenias and decline in serum ferritin levels, underscoring the effectiveness of early combined immunosuppression [5,6,11].

Unlike cases associated with subcutaneous panniculitis-like T-cell lymphoma, which often require aggressive chemotherapy or hematopoietic stem cell transplantation, our patient had no clinical or histological features suggestive of malignancy and responded completely to immunosuppressive therapy [12,13]. At follow-up, she remained clinically stable with no recurrence of disease or features suggestive of lymphoproliferative disorder. Long-term follow-up remains essential due to the risk of recurrence and rare progression reported in the literature [6,12].

Overall, this case reinforces established clinicopathological features of CHP and adds evidence supporting scrub typhus and antecedent trauma as potential triggers. The favorable outcome further supports early combined corticosteroid-cyclosporine therapy in severe or rapidly progressive disease [5,6,11-15].

## Conclusions

Cytophagic histiocytic panniculitis is a rare and potentially life-threatening inflammatory panniculitis that requires early recognition and prompt immunosuppressive therapy to prevent systemic complications and improve outcomes. It often presents with nonspecific clinical features and may rapidly progress to systemic involvement and hemophagocytic lymphohistiocytosis if not recognized early. This case underscores the importance of maintaining a high index of suspicion in children presenting with febrile panniculitis and cytopenias, particularly in the presence of identifiable triggers such as trauma and infection.

Early dermatologic evaluation, timely skin biopsy, and the prompt initiation of immunosuppressive therapy are critical to achieving favorable outcomes. The dramatic clinical and hematological response to combined corticosteroid and cyclosporine therapy observed in our patient reinforces the pivotal role of early intervention in preventing disease progression and reducing mortality. Long-term follow-up remains essential due to the risk of recurrence and the rare possibility of progression to lymphoproliferative disorders. This case highlights the importance of close collaboration between dermatology, pediatrics, and pathology for the early diagnosis and management of this rare entity.
